# Supplementary figures and images for: Low Density Lipoprotein Exposure of Plasmacytoid Dendritic Cells Blunts Toll-like Receptor 7/9 Signaling via NUR77
Source: Biomedicines. 2022 May 17;10(5):1152. doi: 10.3390/biomedicines10051152 (PMC9139034; doi:10.3390/biomedicines10051152)

**Supplemental Figure S1.**

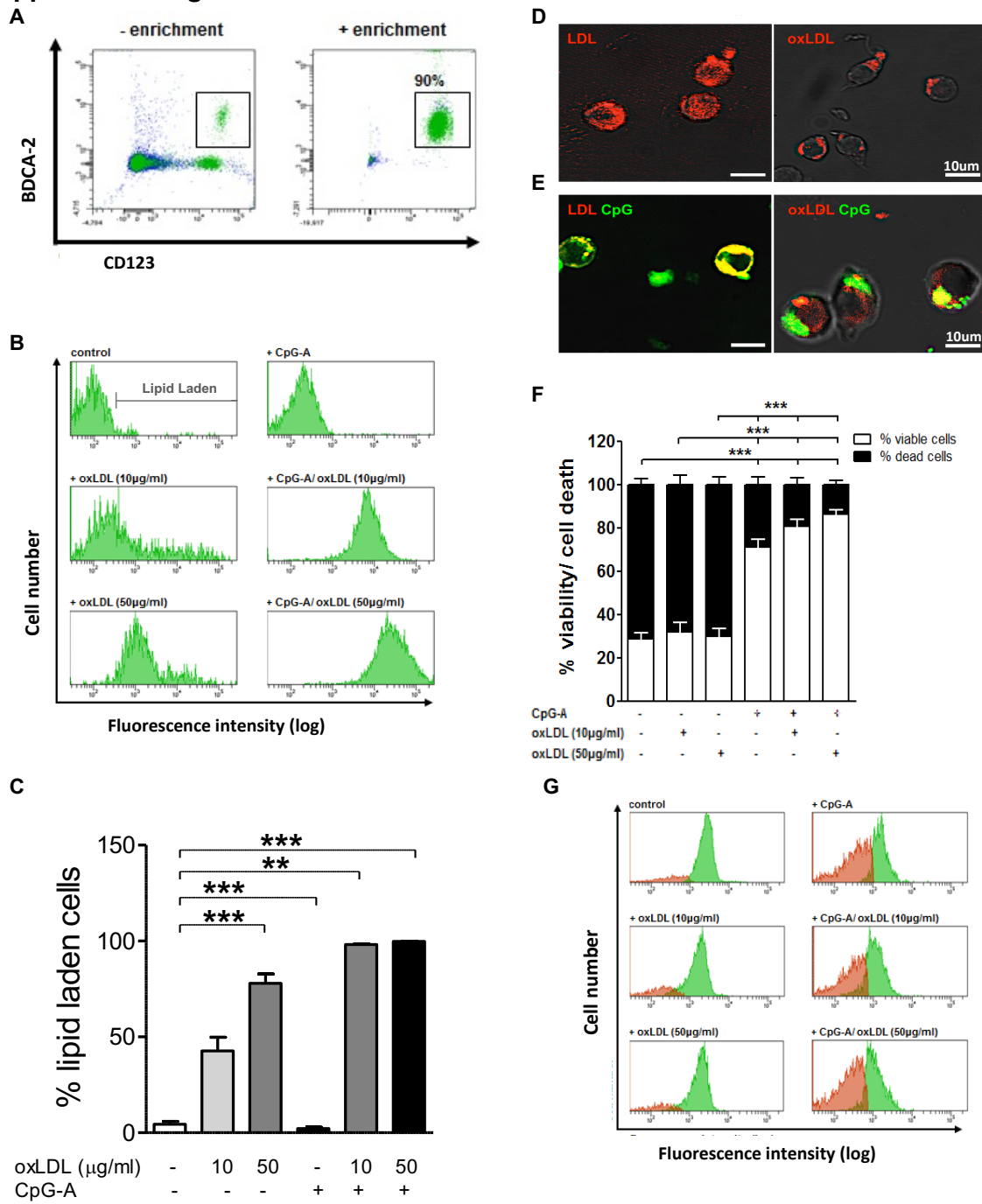

**Supplemental Figure S2.**

**A**

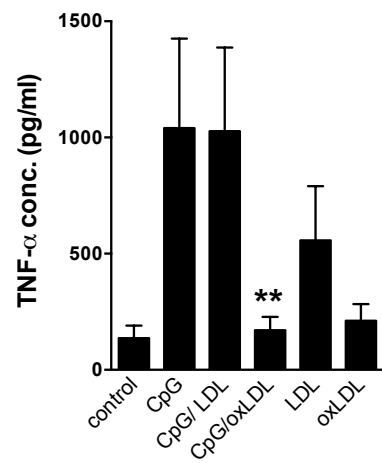

**B**

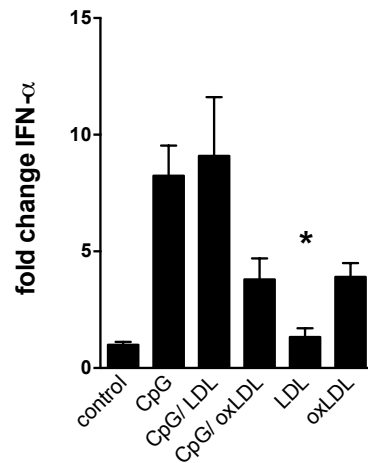

**A**

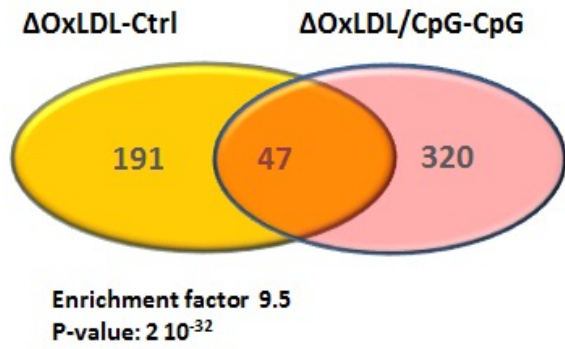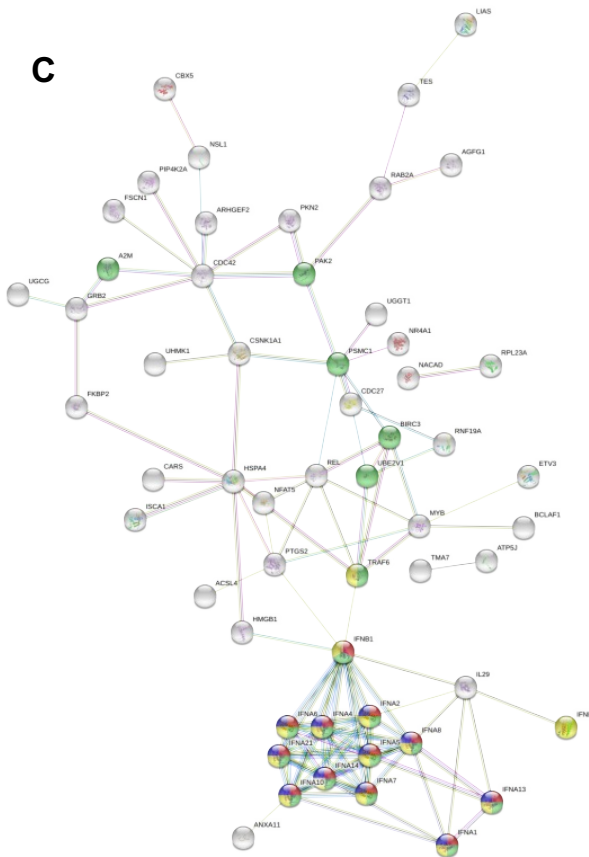

# B

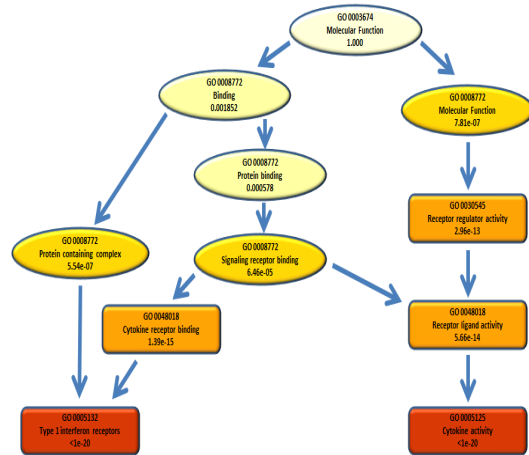

D

[illegible]

## E

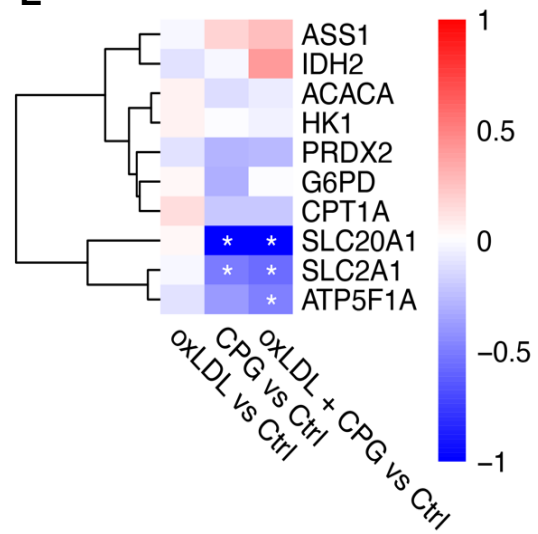

Supplement: Supplementary file 1 [file biomedicines-10-01152-s001.zip › biomedicines-1619263-supplementary.pdf]
